# Supplementary material for: Topological charge-entropy scaling in kagome Chern magnet TbMn6Sn6
Source: Nat Commun. 2022 Mar 7;13:1197. doi: 10.1038/s41467-022-28796-6 (PMC8901788; doi:10.1038/s41467-022-28796-6)
Supplement: Supplementary file 1 — Supplementary Information [file 41467_2022_28796_MOESM1_ESM.pdf]

Supplementary information for

Topological charge-entropy scaling in kagome Chern magnet $TbMn_6Sn_6$

Xitong Xu[1,2], Jia-Xin Yin[3], Wenlong Ma[2], Hung-Ju Tien[4,5], Xiao-Bin Qiang[6], P. V. Sreenivasa Reddy[4], Huibin Zhou[2], Jie Shen[1,7], Hai-Zhou Lu[6], Tay-Rong Chang[4,5,8], Zhe Qu[1,7,9*] & Shuang Jia[2,10,11*]

[1]Anhui Key Laboratory of Condensed Matter Physics at Extreme Conditions, High Magnetic Field Laboratory, Hefei Institutes of Physical Science, Chinese Academy of Sciences, Hefei, Anhui 230031, China

[2]International Center for Quantum Materials, School of Physics, Peking University, Beijing 100871, China

[3]Beijing National Laboratory for Condensed Matter Physics, Institute of Physics, Chinese Academy of Sciences, Beijing 100190, China

[4]Department of Physics, National Cheng Kung University, Tainan 701, Taiwan

[5]Center for Quantum Frontiers of Research and Technology (QFort), Tainan 701, Taiwan

[6]Department of Physics and Shenzhen Institute for Quantum Science and Engineering, Southern University of Science and Technology, Shenzhen 518055, China

[7]Science Island Branch of Graduate School, University of Science and Technology of China, Hefei, Anhui 230026, China

[8]Physics Division, National Center for Theoretical Sciences, Taipei 10617, Taiwan

[9]CAS Key Laboratory of Photovoltaic and Energy Conservation Materials, Hefei Institutes of Physical Science, Chinese Academy of Sciences, Hefei, Anhui 230031, China

[10]Interdisciplinary Institute of Light-Element Quantum Materials and Research Center for Light-Element Advanced Materials, Peking University, Beijing 100871, China

[11]CAS Center for Excellence in Topological Quantum Computation, University of Chinese Academy of Sciences, Beijing 100190, China

*E-mail: zhequ@hmfl.ac.cn; gwljiashuang@pku.edu.cn

Notes 1. Magnetic Properties of $TbMn_6Sn_6$

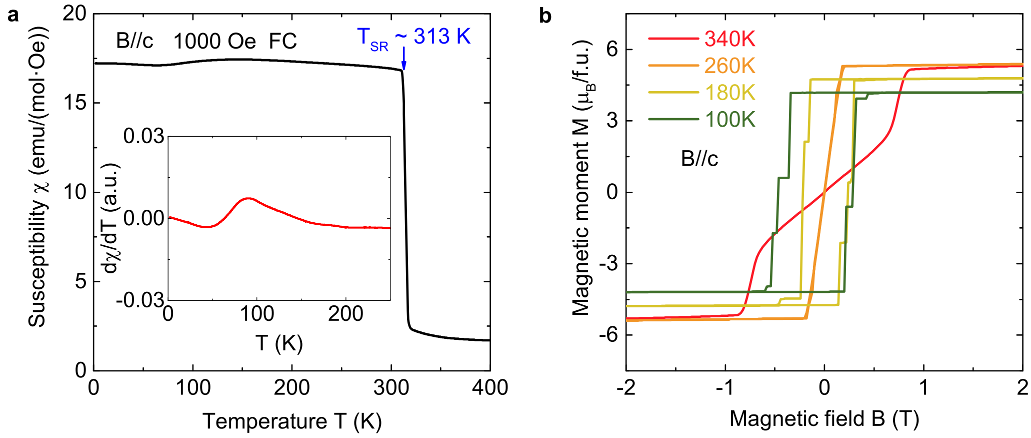

Fig. S1. Magnetic properties of $TbMn_6Sn_6$. a, Magnetic susceptibility during the field cooling process. External field of 1000 Oe is applied along the c direction. Inset shows the temperature derivative of the susceptibility, suggesting a magnetic anomaly at around 100 K. **b,** Field dependence of magnetization at representative temperatures.

Notes 2. Topological Charge-Entropy relations

2.1 Two dimensional massive Dirac model

For the 2D massive Dirac model

$$H = \gamma(k_x\sigma_x + k_y\sigma_y) + (\Delta/2)\sigma_z,$$

the eigen energies can be directly obtained as

$$\varepsilon(k)_\pm = \pm\sqrt{\gamma^2(k_x^2 + k_y^2) + (\Delta/2)^2}.$$

The Berry curvatures for $\pm$ bands are

$$\Omega_\pm = i\nabla_R \times \langle\psi_\pm|\nabla\psi_\pm\rangle = \mp\frac{1}{2r^2}\hat{r},$$

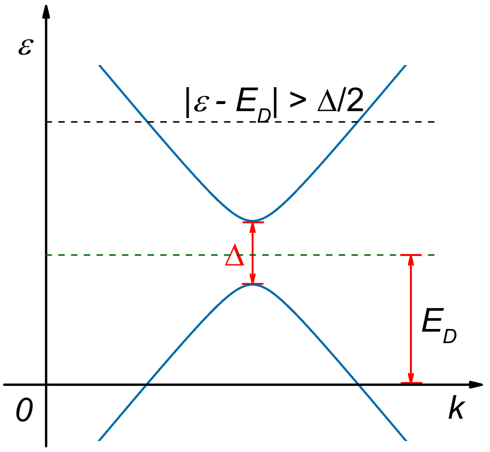

Fig. S2. Band dispersion of the Chern-gapped Dirac model. E_D is the energy of Dirac cone measured from the chemical potential, and we set chemical potential $\mu = 0$.

where $\psi_\pm$ are the eigen states, $R \equiv (r, \theta, \phi)$ represents the spherical coordinate of parameter space, and $r = \sqrt{k_x^2 + k_y^2 + (\Delta/2\gamma)^2}$.

For our purpose, hereafter we consider only the $-$ band as shown in Fig. S2. The anomalous Hall conductivity at $T = 0$ is

$$\sigma_{xy}^A(\varepsilon) = \frac{-e^2}{\hbar} \int \frac{d^2\boldsymbol{k}}{(2\pi)^2} f_0 \Omega_-^z = \frac{e^2}{h} \frac{\Delta}{\sqrt{4\gamma^2 k_f^2 + \Delta^2}},$$

which can be written as

$$\sigma_{xy}^A(\varepsilon) = \frac{e^2}{h} \frac{\Delta}{\sqrt{4\gamma^2 k_f^2 + \Delta^2}} = \begin{cases} \frac{e^2}{h} \frac{\Delta/2}{|\varepsilon - E_D|} & , \quad |\varepsilon - E_D| > \Delta/2 \\ \frac{e^2}{h} & , \quad |\varepsilon - E_D| < \Delta/2. \end{cases}$$

Additionally, for the $T \neq 0$ case we have

$$\begin{aligned} \sigma_{xy}^A &= \frac{-e^2}{\hbar} \int \frac{d^2\boldsymbol{k}}{(2\pi)^2} \Omega_-^z f_0 = \frac{-e^2}{\hbar} \int \frac{d^2\boldsymbol{k}}{(2\pi)^2} \int d\varepsilon \Omega_-^z \left(-\frac{\partial f_0}{\partial \varepsilon}\right) f_0 \\ &\approx \frac{-e^2}{\hbar} \int \frac{d^2\boldsymbol{k}}{(2\pi)^2} \int_{\varepsilon_k}^{\infty} d\varepsilon \Omega_-^z \left(-\frac{\partial f_0}{\partial \varepsilon}\right). \end{aligned}$$

The general coefficient can thus be expressed as

$$\mathcal{C}_n = \frac{1}{\hbar} \int \frac{d^2\boldsymbol{k}}{(2\pi)^2} \int_{\varepsilon_k}^{\infty} d\varepsilon \Omega_-^z \left(\frac{\varepsilon - \mu}{k_B T}\right)^n \left(-\frac{\partial f_0}{\partial \varepsilon}\right).$$

The anomalous thermoelectric Hall conductivity and the anomalous thermal Hall conductivity can be formulated in terms of $\mathcal{C}_n$

$$\alpha_{xy}^A = -k_B e \mathcal{C}_1, \; \kappa_{xy}^A = -k_B^2 T \mathcal{C}_2.$$

2.2 Berry Curvature Effect at Finite Temperature

Using the Sommerfeld expansion,

$$\int Q(\varepsilon)\left(-\frac{\partial f_0}{\partial \varepsilon}\right) d\varepsilon \approx Q(\mu) + \frac{\pi^2}{6}(k_B T)^2 Q''(\varepsilon)|_{\varepsilon=\mu} + \frac{7\pi^4}{360}(k_B T)^4 Q''''(\varepsilon)|_{\varepsilon=\mu} + \mathcal{O}(T^6),$$

the anomalous Hall conductivity can be written as

$$\begin{aligned}
\sigma_{xy}^A &= \frac{-e^2}{\hbar}\int d\varepsilon \int \frac{d^2\boldsymbol{k}}{(2\pi)^2}\Omega_-^z f_0 \left(-\frac{\partial f_0}{\partial \varepsilon}\right) \\
&= \frac{-e^2}{\hbar}\int d\varepsilon Q(\varepsilon)\left(-\frac{\partial f_0}{\partial \varepsilon}\right) \\
&\approx \frac{-e^2}{\hbar} Q(\mu) + \mathcal{O}(T^2)
\end{aligned}$$

where

$$\begin{aligned}
Q(\varepsilon) &= \int \frac{d^2\boldsymbol{k}}{(2\pi)^2}\Omega_-^z f_0 = \frac{-\Delta}{2\pi\sqrt{4\gamma^2 k_f^2 + \Delta^2}} \\
&= \begin{cases} \dfrac{-\Delta/2}{2\pi\,|\varepsilon - E_D|} & , \quad |\varepsilon - E_D| > \Delta/2 \\ \dfrac{-1}{2\pi} & , \quad |\varepsilon - E_D| < \Delta/2. \end{cases}
\end{aligned}$$

The anomalous thermal Hall conductivity is written as

$$\begin{aligned}
\kappa_{xy}^A &= \frac{-k_B^2 T}{\hbar}\int d\varepsilon \left(\frac{\varepsilon-\mu}{k_B T}\right)^2 \int \frac{d^2\boldsymbol{k}}{(2\pi)^2}\Omega_-^z f_0 \left(-\frac{\partial f_0}{\partial \varepsilon}\right) \\
&= \frac{-1}{T\hbar}\int d\varepsilon(\varepsilon-\mu)^2 Q(\varepsilon)\left(-\frac{\partial f_0}{\partial \varepsilon}\right) \\
&\approx \frac{-1}{T\hbar}\left[\frac{\pi^2}{6}(k_B T)^2 2Q(\mu) + \frac{7\pi^4}{360}(k_B T)^4 12 Q''(\mu)\right] + \mathcal{O}(T^6) \\
&= \frac{-k_B^2 T}{\hbar}\frac{\pi^2}{3}Q(\mu) - \frac{k_B^4 T^3}{\hbar}\frac{7\pi^4}{30}Q''(\mu)
\end{aligned}$$

Thus, the Lorentz number reads

$$L^A_{xy} = \frac{\kappa^A_{xy}}{\sigma^A_{xy} T} = L_0 + \frac{21}{10} e^2 L_0^2 T^2 \frac{Q''(\mu)}{Q(\mu)} = L_0 + \frac{21}{5} L_0^2 T^2 \frac{1}{E_D^2},$$

where $L_0 = \pi^2 k_B^2 / 3e^2$ is the Sommerfeld value.

For the anomalous Nernst conductivity induced by the Berry curvature,

$$\begin{aligned}\alpha^A_{xy} &= -\frac{k_B e}{\hbar} \int d\varepsilon \frac{\varepsilon - \mu}{k_B T} \int \frac{d^2\boldsymbol{k}}{(2\pi)^2} \Omega^z_z f_0 \left(-\frac{\partial f_0}{\partial \varepsilon}\right) \\ &= \frac{-e}{T\hbar} \int d\varepsilon (\varepsilon - \mu) Q(\varepsilon) \left(-\frac{\partial f_0}{\partial \varepsilon}\right) \\ &\approx \frac{-e}{T\hbar} \frac{\pi^2}{6} (k_B T)^2 2Q'(\mu) + \mathcal{O}(T^4) \\ &= -\frac{e k_B^2 T}{\hbar} \frac{\pi^2}{3} Q'(\mu)\end{aligned}$$

Therefore

$$\frac{\alpha^A_{xy}}{\sigma^A_{xy}} = e L_0 T \frac{Q'(\mu)}{Q(\mu)} = e L_0 T \frac{1}{E_D}$$

In short, we have the following two topological charge-entropy relations,

$$\kappa^A_{xy} / \sigma^A_{xy} = L_0 T \left[1 + \eta \left(\frac{k_B T}{E_D} \right)^2 \right], \ \eta = \frac{7\pi^2}{5} \tag{S1}$$

$$\alpha^A_{xy} / \sigma^A_{xy} = \frac{\pi^2}{3} \frac{k_B}{e} \frac{k_B T}{E_D} \tag{S2}$$

2.3 The Role of Chern Dirac Band in $TbMn_6Sn_6$

In previous works[1,2] it has been proven explicitly that in $TbMn_6Sn_6$ the Berry curvature from the Dirac hole band dominates σ^A_{xy}, or equivalently, $\tilde{\sigma}_{xy}(\varepsilon)$ in Eq. 7 in the main text. According to the analysis in the previous section, it can be readily shown that $\alpha^A_{xy} \propto \tilde{\sigma}'_{xy}(\varepsilon)$, while $\kappa^A_{xy} \propto \tilde{\sigma}_{xy}(\varepsilon) + \eta \tilde{\sigma}''_{xy}(\varepsilon)$. These two formulas indicate that the Berry curvature singularity from the Dirac hole band also prevails over trivial bands in both α^A_{xy} and κ^A_{xy}.

The neglecting contribution of the trivial bands is also seen from our analysis of the topological scaling relations (Eqs.S1&S2) for the Chern-gapped Dirac model. Since the violation of the Wiedemann Franz law is stemming from the term proportional to $(k_B T/E_D)^2$, such a violation should not exist for a generic band with some Berry curvatures. Our observations in experiment support our hypothesis that the contribution from the generic band is small in the topological charge-entropy scaling.

2.4 The Role of Skew Scattering in the Scaling Relations

In Fig. 2d in the main text, there is an upturn feature of the anomalous Hall conductivity (AHC) σ_{AH} at low temperatures. In order to understand this change and its influence on the three anomalous effects at low temperatures, we measured several different batches of samples. We noticed that they show slightly different AHC at the lowest temperatures, but the high-temperature data is very close. Below we show the σ_{AH} versus σ_{xx} for two typical samples (S1 in the main text and S2 from another batch) in Fig. S3.

It is well-known that the AHC σ_{AH} can be distinguished into three regions[4] according to its longitudinal conductivity σ_{xx}: The high conductivity region where the skew scattering mechanism dominates ($\sigma_{AH} \sim \sigma_{xx}$); the good metal region where intrinsic mechanism dominates ($\sigma_{AH} \sim const$); and the dirty metal region where localized hopping plays a role ($\sigma_{AH} \sim \sigma_{xx}^{1.6\sim1.8}$). Theoretical analyses by Onoda et al.[5] also shows no well-defined plateau in the good metal region when a small impurity potential is considered.

$TbMn_6Sn_6$ shows overall correspondence to this scenario. For samples S1 and S2, the conductivity roughly lies in the good metal region. At low temperatures, the conductivity is high and there is a crossover behavior to skew scattering region seen as an enhancing tendency of total σ_{AH} with σ_{xx}. We calculated the corresponding σ_{AH}^{Skew} for these two samples. As shown

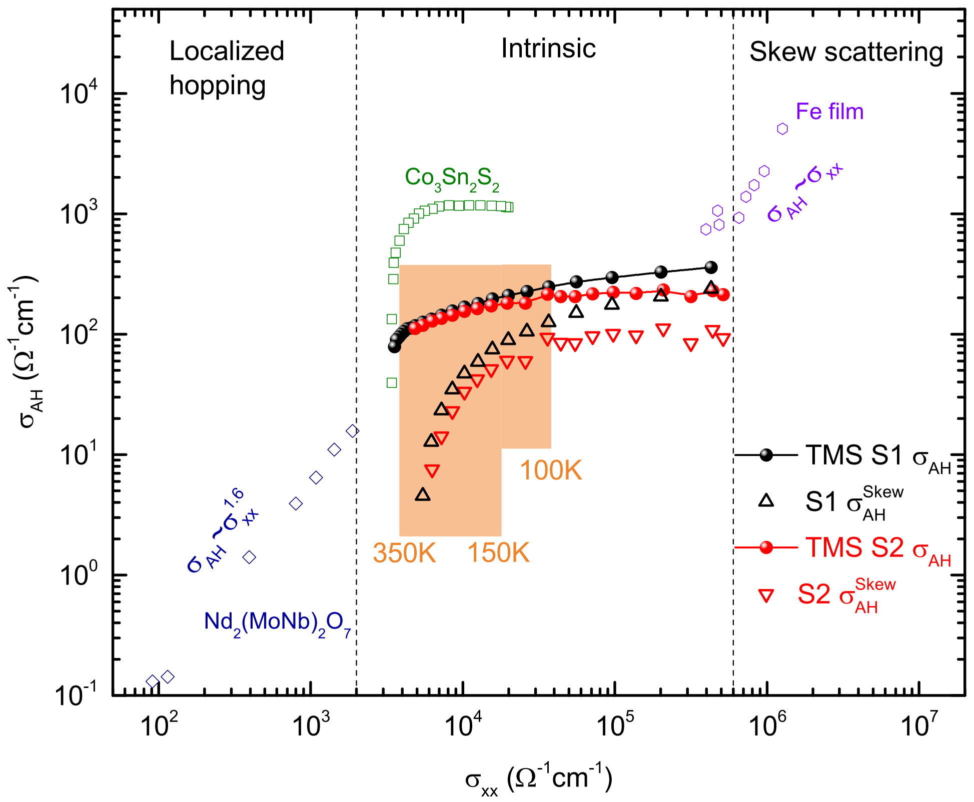

Fig. S3. Scaling of the anomalous Hall conductivity σ_{AH} with longitudinal conductivity σ_{xx}. Three regions are usually distinguished, including the localized hopping region where $\sigma_{AH} \sim \sigma_{xx}^{1.6\sim1.8}$, the intrinsic region where $\sigma_{AH} \sim const$, and the skew scattering region where $\sigma_{AH} \sim \sigma_{xx}$. The data for $Nd_2(MoNb)_2O_7$, $Co_3Sn_2S_2$ and Fe films are reproduced from Ref.[3] as examples. The σ_{AH} and the extracted extrinsic contribution σ_{AH}^{Skew} of two representative $TbMn_6Sn_6$ samples (S1 and S2) are shown. For our interested temperature range (shaded area), the intrinsic contributions play a dominant role.

in Fig. S3, σ_{xy}^{Skew} accounts for around 50% (40%) of total σ_{AH} in sample S1(S2) at 100 K, and this ratio quickly decreases to less than 10% at 250 K and is neglecting small at 300 K. We conclude that the intrinsic contribution plays a dominating role in the high temperature range. Note the convergence of the σ_{AH} for different samples when temperature is above 150 K, which unveils that the intrinsic part of AHC is the same for different samples while the extrinsic part can be sample-dependent at low temperature. The high temperature part is also the range where the experimental observed ratios $\alpha_{xy}^A/\sigma_{xy}^A$ and $\kappa_{xy}^A/\sigma_{xy}^A$ best fit our topological charge-entropy scaling relations in Fig. 3c&d.

We now show that extrinsic contribution at low temperatures will not influence our conclusion. It has been proven[6–8] that in the absence of inelastic scattering, the Mott relation and Wiedemann Franz law generally hold for transport coefficients in the low temperature limit, which has also been verified in experiments. Specifically, this means that $\kappa_{xy}^{A-skew}/\sigma_{xy}^{A-skew}$ should be equal to $\kappa_{xy}^A/\sigma_{xy}^A$ and obeys the Wiedemann Franz law as well at low temperatures. On the other hand, our topological charge-entropy relation on $\kappa_{xy}^A/\sigma_{xy}^A = L_0 T\left(1+\eta\left(\frac{k_BT}{E_D}\right)^2\right)$ (Eq.S1) is a modified version of the Wiedemann Franz law considering higher order contributions from Chern Dirac topology at finite temperatures. These give interesting interpretations that our scaling relation Eq.S1 can be applied to a wide temperature range: at low temperatures the ratio is close to L_0T even in the presence of extrinsic skew scattering effect; at temperatures in the order of E_D/k_B, Berry curvature modification from the Chern Dirac cone will result in a violation of the Wiedemann Franz law. In Fig. S4b we show this scaling in both samples S1 and S2 with different skew scattering strength, and they indeed share similar profiles.

Similar deduction can also be done for $\alpha_{xy}^A/\sigma_{xy}^A$ assuming the extrinsic part scales with the intrinsic one. It can be readily seen in Fig. S4a that the experimental data for the two samples also converge in the high temperature parts. The small deviation below 200 K may arise from

the different energy dependence between $\sigma_{xy}^{A-skew}(\varepsilon)$ and $\sigma_{xy}^{A-int}(\varepsilon)$.

Based on the discussions above, we conclude that: 1, the intrinsic contribution dominates in the interested temperature range we investigate, and 2, the presence of extrinsic contribution at low temperature will not influence our conclusion.

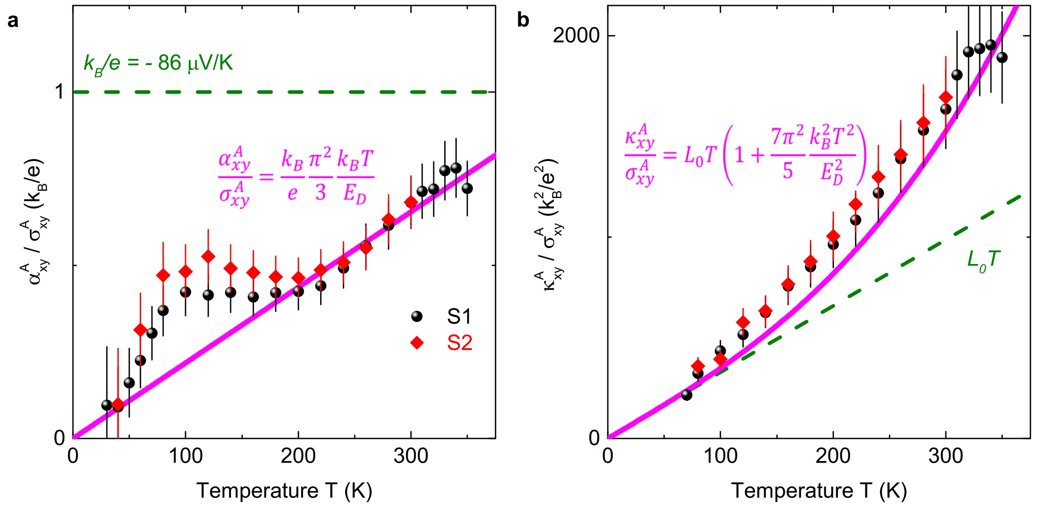

Fig. S4. The topological charge-entropy scaling relations for $TbMn_6Sn_6$. Samples S1 and S2 are from different growth batches. The error bars reflect the uncertainty in determining the samples' geometric factor and the low temperature anomalous Nernst signals.

Finally we briefly discuss the deviation of σ_{AH} from our polynomial fitting in Fig. 3a inset. According to Ref.[4], the decrease of σ_{AH} near the dirty metal region is due to the influence of finite-lifetime broadening on the intrinsic contribution σ_{xy}^{A-int}. This leads to a decay of σ_{AH} faster than linearity with respect to σ_{xx}, which has been observed in a variety of materials, including recent $Co_3Sn_2S_2$[9], Mn_3Ge/Sn[10,11]. Previous studies have suggested that the finite-lifetime broadening in this region has same influence on the anomalous transport coefficients, which means the ratios $\alpha_{xy}^A/\sigma_{xy}^A$ and $\kappa_{xy}^A/\sigma_{xy}^A$ retain the same Berry curvature effects[10,12].

In our $TbMn_6Sn_6$ samples, when temperature is above 320 K, there is also a decrease of σ_{AH}. We point out that the deviation of $\kappa_{xy}^A/\sigma_{xy}^A$ from T-linearity occurs well below this temperature and shows a maximal slope already at around 300 K. Due to the large torque from

in-plane magnetism above 320 K, the samples crash easily in practice and it has been hard for us to measure α_{xy}^A and κ_{xy}^A above 350 K. The effect of the quasiparticle broadening at higher temperatures will be an interesting research effort in future.

Notes 3. Low Temperature Nernst Effect

The low temperature Nernst effect in $TbMn_6Sn_6$ is shown in Fig. S5. The ANE in $TbMn_6Sn_6$ quickly drops below 100 K and the value is as small as 0.04 μV/K at 50 K. Below this temperature, the ANE can be hardly resolved and the normal Nernst effects dominate. The QOs in Nernst signals are similar to those in the Seebeck measurements.

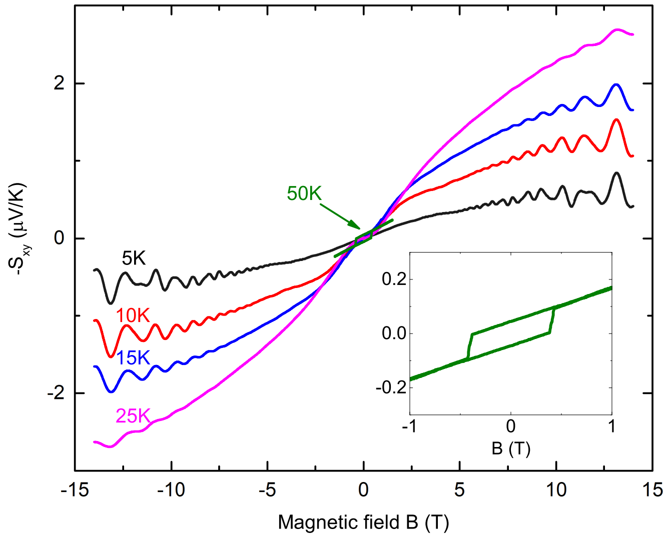

Fig. S5. Low temperature Nernst effect in $TbMn_6Sn_6$. Inset: Zoom-in of the small anomalous Nernst loop at 50 K.

Supplementary Notes 4. Thermoelectric Performance

The ANE in ordinary ferromagnets is known to be roughly proportional to the magnetization[13], $|S_{xy}^A| = |Q_s|\mu_0 M$, where $|Q_s|$ is the anomalous Nernst coefficient ranging between 0.05 and

1 μV/KT. As shown in Fig.S6 below, the ANE in $TbMn_6Sn_6$ greatly surpasses this empirical law. Due to the ferrimagnetic order and the Chern Dirac topology, $TbMn_6Sn_6$ exhibits a large ANE (-2.4 μV/K) comparable to Mn_3Ge, $Co_3Sn_2S_2$ and Co_2MnGa, whereas the magnetization is much smaller than the ferromagnet Co_2MnGa. This makes $TbMn_6Sn_6$ promising in developing efficient, large-scale, flexible ANE thermopiles[14].

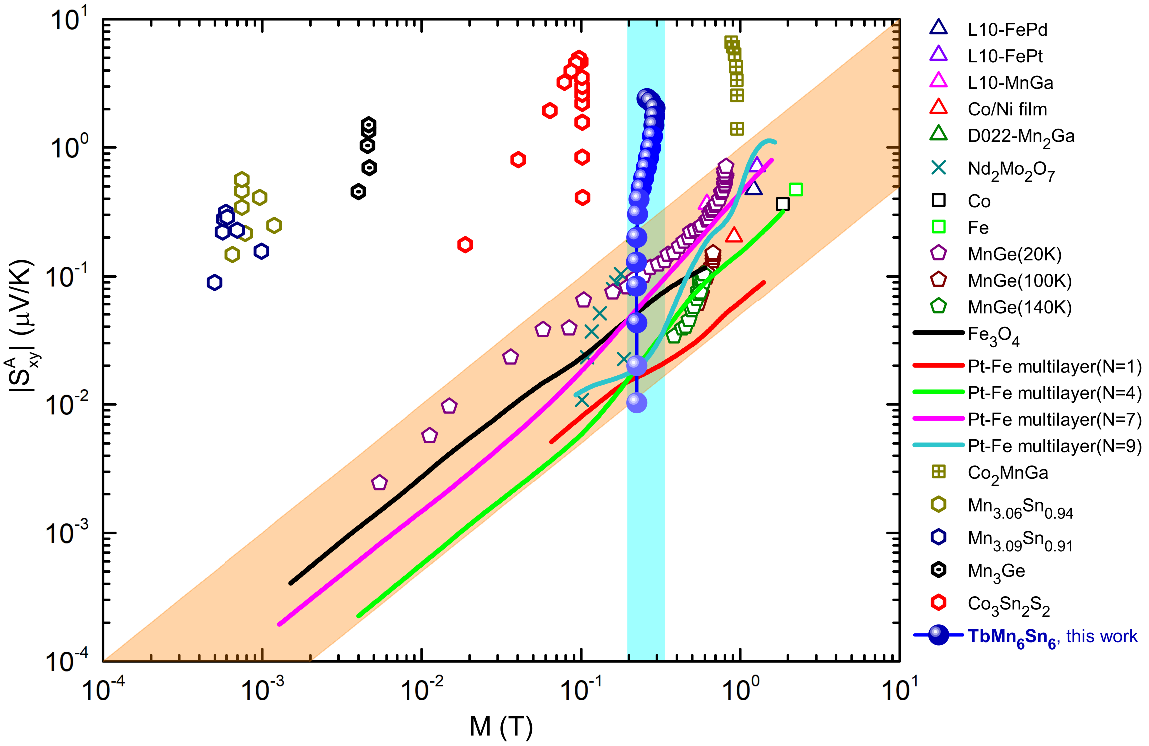

Fig. S6. Anomalous Nernst effect scaled against magnetization. Magnetization dependence of the anomalous Nernst effect for ferromagnetic metals, antiferromagnets Mn_3(Sn/Ge), and ferrimagnet $TbMn_6Sn_6$. The orange shaded area indicates the linear relation $|S^A_{xy}| = |Q_s|\mu_0 M$ where $|Q_s|$ is the anomalous Nernst coefficient ranging between 0.05 and 1 μV/KT. Data are reproduced based on Refs.[13,15,16].

Compared to the ANE, the anomalous thermal Hall effect (ATHE) is less studied. From the data available, we notice that the room temperature ATHE in $TbMn_6Sn_6$ (0.155 W/Km) is much larger than those in Mn_3Ge (0.015 W/Km, 300 K)[10] and Fe_3Sn_2 (0.09 W/Km, 300 K)[17]. $Co_3Sn_2S_2$ possesses a large ATHE (0.2 W/Km) at 150 K[18], but it becomes paramagnetic above

180 K. The ATHE in Ni is in the order of 0.4 W/Km at 300 K[19], but there is also a large normal thermal Hall contribution. The large room temperature ATHE in $TbMn_6Sn_6$ can therefore be a candidate for applications like magnetic controlling of thermal currents[20].

The thermoelectric figure of merit is formulated as

$$ZT = \frac{\sigma S^2 T}{\kappa}$$

where S can be one of S_{xx}, S_{xy}, S^A_{xy} ($S^A_{xy} = \rho_{xx}\alpha^A_{xy} + \rho^A_{xy}\alpha_{xx}$), corresponding to Seebeck, Nernst, anomalous Nernst figure of merit, and σS^2 is the power factor PF. For our purposes, we are interested in the Seebeck and anomalous Nernst figure of merit, since the Nernst effect is dominated by the anomalous part around room temperature. In Fig.S7 we show the figures of merit and power factors of $TbMn_6Sn_6$. The Seebeck and anomalous Nernst ZT increases with temperature, and the former is much larger than the latter, reaching 0.012 at 350 K. The PF of the Seebeck one is also larger, being around 3 $\mu\mathrm{W/K^2cm}$ at 300 K.

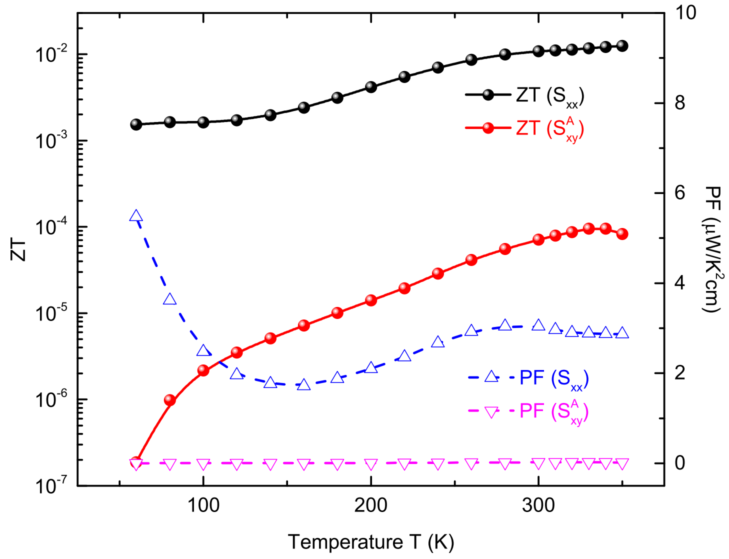

Fig. S7. Thermoelectric Performances. Seebeck and anomalous Nernst figures of merit and corresponding power factor in $TbMn_6Sn_6$.

It is hard to give a general prediction on the magnitude of ZT or PF, considering σ, S and

κ depend sensitively on carrier mobilities and phonon process aside from the Berry curvature effect. Nevertheless, we can give some guidance based on our scaling relations.

In our previous work[2], we have shown that in the ferrimagnet RMn_6Sn_6 systems, due to the coupling between 4f moments and 3d electrons of Mn kagome lattice, the Chern Dirac cones gap Δ and energy E_D roughly follow a rough dG and $\sqrt{dG}$ relation, where dG is the de Gennes factor of 4f moments. Combined with Eq.S1, we have

$$\sigma_A \propto \frac{\Delta}{E_D} \propto \sqrt{dG} \tag{S3}$$

$$\alpha_A \propto \frac{\Delta}{E_D^2} T \propto const \times T \tag{S4}$$

This gives an interesting prediction that at a fixed temperature like 300 K, α_A across different R substitution in RMn_6Sn_6 would change little. In order to enhance α_A, electron doping on the Mn or Sn sites to break the $\sqrt{dG}$ dependence of E_D seems necessary. In Ref.[1] it has been shown that partial Fe substitution on Mn site can effectively tunes the Fermi level closer to the Chern Dirac gap. We propose that this would simultaneously enhance the anomalous thermoelectric Hall conductivity α_A.

References

[1] Yin, J.-X. et al. Quantum-limit Chern topological magnetism in $TbMn_6Sn_6$. *Nature* **583**, 533–536 (2020).

[2] Ma, W. et al. Rare earth engineering in RMn_6Sn_6 (R = Gd - Tm, Lu) topological kagome magnets. *Phys. Rev. Lett.* **126**, 246602 (2021).

[3] Yang, S.-Y. et al. Giant, unconventional anomalous Hall effect in the metallic frustrated magnet candidate, KV_3Sb_5. *Sci. Adv.* **6**, eabb6003 (2020).

[4] Nagaosa, N. et al. Anomalous Hall effect. *Rev. Mod. Phys.* **82**, 1539–1592 (2010).

[5] Kovalev, A. A. et al. Transport theory for disordered multiple-band systems: Anomalous Hall effect and anisotropic magnetoresistance. *Phys. Rev. B* **79**, 195129 (2009).

[6] Smrcka, L. and Streda, P. Transport coefficients in strong magnetic fields. *J. Phys. C: Solid State Phys.* **10**, 2153–2161 (1977).

[7] Onoda, S., Sugimoto, N. and Nagaosa, N. Quantum transport theory of anomalous electric, thermoelectric, and thermal Hall effects in ferromagnets. *Phys. Rev. B* **77**, 165103 (2008).

[8] Qin, T., Niu, Q. and Shi, J. Energy magnetization and the thermal Hall effect. *Phys. Rev. Lett.* **107**, 236601 (2011).

[9] Liu, E. et al. Giant anomalous Hall effect in a ferromagnetic kagome-lattice semimetal. *Nat. Phys.* **14**, 1125–1131 (2018).

[10] Xu, L. et al. Finite-temperature violation of the anomalous transverse Wiedemann-Franz law. *Sci. Adv.* **6**, eaaz3522 (2020).

[11] Nakatsuji, S., Kiyohara, N. and Higo, T. Large anomalous Hall effect in a non-collinear antiferromagnet at room temperature. *Nature* **527**, 212–215 (2015).

[12] Li, X. et al. Anomalous Nernst and Righi-Leduc effects in Mn_3Sn: Berry curvature and entropy flow. *Phys. Rev. Lett.* **119**, 056601 (2017).

[13] Ikhlas, M. et al. Large anomalous Nernst effect at room temperature in a chiral antiferromagnet. *Nat. Phys.* **13**, 1085–1090 (2017).

[14] Sakai, A. et al. Iron-based binary ferromagnets for transverse thermoelectric conversion. *Nature* **581**, 53–57 (2020).

[15] Sakai, A. et al. Giant anomalous Nernst effect and quantum-critical scaling in a ferromagnetic semimetal. *Nat. Phys.* **14**, 1119–1124 (2018).

[16] Yang, H. et al. Giant anomalous Nernst effect in the magnetic Weyl semimetal $Co_3Sn_2S_2$. *Phys. Rev. Materials* **4**, 024202 (2020).

[17] Zhang, H., Xu, C. Q. and Ke, X. Topological Nernst effect, anomalous Nernst effect, and anomalous thermal Hall effect in the Dirac semimetal Fe_3Sn_2. *Phys. Rev. B* **103**, L201101 (2021).

[18] Ding, L. et al. Quantum oscillations, magnetic breakdown and thermal Hall effect in $Co_3Sn_2S_2$. *J. Phys. D: Appl. Phys.* **54**, 454003 (2021).

[19] Onose, Y., Shiomi, Y. and Tokura, Y. Lorenz number determination of the dissipationless nature of the anomalous Hall effect in itinerant ferromagnets. *Phys. Rev. Lett.* **100**, 016601 (2008).

[20] Ideue, T. et al. Giant thermal Hall effect in multiferroics. *Nat. Mater.* **16**, 797–802 (2017).
